# SARS-CoV-2 RNA and Supermarket Surfaces: A Real or Presumed Threat?

**DOI:** 10.3390/ijerph18179404

**Published:** 2021-09-06

**Authors:** Giuseppina Caggiano, Francesco Triggiano, Francesca Apollonio, Giusy Diella, Marco Lopuzzo, Marilena D’Ambrosio, Fabrizio Fasano, Pasquale Stefanizzi, Giovanni Trifone Sorrenti, Pantaleo Magarelli, Domenico Pio Sorrenti, Vincenzo Marcotrigiano, Osvalda De Giglio, Maria Teresa Montagna

**Affiliations:** 1 Department of Biomedical Science and Human Oncology—Hygiene Section, University of Bari Aldo Moro, Piazza G. Cesare 11, 70124 Bari, Italy; francesco.triggiano@uniba.it (F.T.); francesca.apo@libero.it (F.A.); giusy.diella@uniba.it (G.D.); marcolopuzzo@gmail.com (M.L.); marilena.dambrosio@uniba.it (M.D.); fabrizio.fasano1979@libero.it (F.F.); pasquale.stefanizzi@uniba.it (P.S.); osvalda.degiglio@uniba.it (O.D.G.); mariateresa.montagna@uniba.it (M.T.M.); 2 Department of Prevention, Food Hygiene and Nutrition Service, Local Health Unit BT, Corso M. R. Imbriani 138, 76125 Trani, Italy; giovanni.sorrenti@aslbat.it (G.T.S.); pantaleo.magarelli@aslbat.it (P.M.); d.sorrenti2@studenti.uniba.it (D.P.S.); vincenzo.marcotrigiano@aslbat.it (V.M.)

**Keywords:** SARS-CoV-2, supermarket, handles, scales, keyboards, surfaces, COVID-19, shopping trolley handle, refrigeration system handle

## Abstract

Severe Acute Respiratory Syndrome Coronavirus 2 (SARS-CoV-2) emerged in March 2020 in Italy, leading to the pandemic of coronavirus disease 2019 (COVID-19) that continues to cause high global morbidity and mortality in human populations. Numerous studies have focused on the spread and persistence of the virus in the hospital setting. New scientific evidence shows that SARS-CoV-2 is present in different community environments. Although aerosol is one of the main routes of transmission for SARS-CoV-2, indirect contact through virus-contaminated surfaces could also play a key role. The survival and persistence of SARS-CoV-2 on surfaces appear to be influenced by the characteristics of the material, temperature, and humidity. In this study, we investigated the presence of SARS-CoV-2 RNA on surfaces in 20 supermarkets throughout the Apulia region during the lockdown period. We collected a total of 300 swab samples from various surfaces including supermarket scales, trolley handles, refrigerator and freezer handles, and keyboards. In total, 13 (4.3%) surfaces were positive for SARS-CoV-2 RNA contamination, with shopping trolley handles being the most frequently contaminated. This study showed that**** contamination in public spaces can occur, so we remark the importance to adopt adequate preventive measures, including environment ventilation, careful surfaces sanitation, hand hygiene, and correct usage of masks, to reduce the likelihood of virus transmission.

## 1. Introduction

More than a year after the World Health Organization declared the start of the COVID-19 pandemic in March 2020, and despite efforts to eliminate the virus, Severe Acute Respiratory Syndrome Coronavirus 2 (SARS-CoV-2) remains a threat to global health. Belonging to *Coronaviridae* and characterized by positive-sense single-stranded RNA, this virus is responsible for approximately 193,770,895 infections and four million deaths globally as of 19 July 2021, among which 4,312,673 infections and 127,942 deaths have been in Italy [1]. Although there has never been such a huge amount of information acquired about a disease in such a short time, many aspects of SARS-CoV-2 have yet to be investigated [2,3]. The virus can be transmitted directly from person to person, which is the route most likely to result in infection, but also through other routes, including contaminated surfaces. Nevertheless, the role that surfaces have on the spread of the disease remains debated. Despite common detection of SARS-CoV-2 RNA on surfaces, some studies confirm that the risk of transmission via fomites may be low in clinical settings [4,5].

Jones R.M. [6] demonstrated that, when personal protective equipment is not used, droplet and inhalation transmission routes predominate over the contact route, contributing 35%, 57%, and 8.2% of the probability of infection. Some authors report that environmental contamination by SARS-CoV-2 is high in hospital settings [4,5,7,8] but it is not yet well known whether asymptomatic individuals can spread the infection in the same way as patients with coronavirus disease 2019 (COVID-19). Surface contamination by asymptomatic individuals has been demonstrated [9], but data in non-healthcare settings are still minimal. Harvey et al. [10] estimated risk of infection from touching a contaminated surface to be low (less than 5 in 10,000). However, it is suspected that indirect contact through virus-contaminated surfaces may also have a role in transmission [11,12]. Besides, environmental surveillance is an important tool for monitoring infectious disease prevalence, especially diseases with several asymptomatic subjects, so targeted sampling of high-touch surfaces can help with other pandemic surveillance strategies in identifying the passage of infected subjects. 

In Italy, SARS-CoV-2 RNA has been detected in molecular investigations of surfaces in tourist and recreational facilities, such as bathroom door handles, refrigerator handles, handrails, and bar counters [13]. Although molecular investigation can detect the viral genome, viability of the virus has not been demonstrated using these methods. 

The survival and persistence of SARS-CoV-2 on surfaces appear to be conditioned by characteristics of the material as well as temperature and humidity. A recent study [14] showed that UK SARS-CoV-2 strain, England 02/2020 remains viable for longer periods on hydrophobic surfaces (up to seven days) as compared with hydrophilic surfaces (three days). 

The aim of the study was to investigate the presence of SARS-CoV-2 RNA on surfaces in supermarkets to inform which surfaces are most contaminated and require more attention during disinfection procedures.

## 2. Materials and Methods

### 2.1. Study Design

According to decrees of the President of the Republic dated 3 November 2020 and 14 January 2021, regions throughout Italy were categorized into one of four levels—white, yellow, orange, and red—representing the lowest to highest risk of SARS-CoV-2 spread. The risk level in each region is based on parameters identified by the Istituto Superiore di Sanità, including the number of symptomatic patients with COVID-19, hospitalizations, new outbreaks, occupied hospital beds, and deaths owing to COVID-19.

The present study was conducted from April to May 2021, when the Apulia region in Southern Italy was at orange and red levels. At the orange level, people could move freely within their municipality of residence; at the red level, people were required to avoid social interactions and to stay at home. Movement was only allowed for work or health reasons or to buy food and essentials.

**Figure 1:**
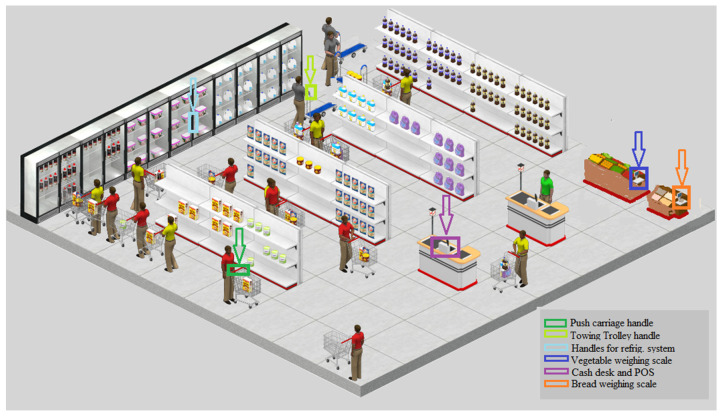
Example diagram of the sampling points. POS, point of sale.

Overall, we analyzed 20 supermarkets in this study, selected from among the most popular in one province of Apulia. We sampled 15 points in each supermarket, chosen from among those considered most representative of surfaces that have a high level of contact with customers’ hands (Figure 1). These sampling points included:

1. Scales (*n* = 20): self-service produce scales and self-service bread scales.
2. Shopping trolley handles (*n* = 120): trolley handles and push trolley handles.
3. Refrigeration system handles (*n* = 80): refrigerator handles (drinks and dairy products) and freezer handles (ice cream, frozen fish and vegetables, and mixed frozen products).
4. Keyboards (*n* = 80): cash register keyboard used by cashiers and POS keyboards used by customers.

### 2.2. Environmental Sampling

Surface sampling was carried out using sterile swabs inserted into plastic tubes (Easy Surface Checking Swab Neutralizing Rinse Solution, NRS; Liofilchem Srl, Roseto degli Abruzzi, TE, Italy) containing 10 mL of transport medium. Sampling was carried out on flat surfaces by sliding and rotating a moistened swab over a standard-sized sampling area (10 × 10 cm); on non-flat surfaces, any available area (e.g., handles) was sampled. Sampling was carried during time slots that were representative of high levels of customer utilization (between 9 a.m. and 12 a.m.). Samples were transported to the laboratory in a special isothermal refrigerator at a controlled temperature (4 °C) and immediately processed. 

### 2.3. Molecular Analysis

To evaluate the method of extraction, amplification/detection, and efficiency recovery, preliminary in vitro studies were conducted on different types of surfaces such as wood, steel, and glass using a strain of Feline Coronavirus (FCoV) type II 25/92, isolated from a cat with infectious peritonitis. The swabs were vortexed for 20 s and transferred to a new 15-mL tube under sterile conditions. We conducted molecular investigation for the presence of SARS-CoV-2 RNA using real-time reverse transcription-polymerase chain reaction (RT-PCR), as documented in the literature and our previous study [13,15]. Nucleic acid was extracted with 5 mL of NRS medium using the NucliSENS miniMAG semi-automatic extraction system with magnetic silica, according to the manufacturer’s instructions (bioMerieux, Marcy-l’Etoile, Lyon, France).

To monitor the quality of the extraction procedure, we used a negative control (i.e., a swab with sterile neutralizer) and positive control (RNA extraction of our process control virus, or FCoV). RNA was resuspended in 100 µL of elution buffer and the extracts were stored at −20 °C. 

For amplification of the ORF-1ab gene (nsp14), a mixture of 25 μL was prepared containing 5 µL of RNA for each sample; 12.5 µL of 2X Reaction Buffer supplied with AgPath-ID™ One-Step RT-PCR Reagents (Applied Biosystems, Thermo Fisher Scientific, MA, USA); 1 µL of 25X RT-PCR enzyme mix; 1 µL of forward primer (12.5 µM); 1 µL of reverse primer (22.5 µM); 1 mL of probe (6.25 µM); 1.83 µL of nuclease-free water (not DEPC-treated); and 1.67 µL Real-Time PCR Detection Enhancer (Applied Biosystems). The primer and probe sequences used were as follows: CoV-2-F/ACA TGG CTT TGA GTT GAC ATC T; CoV-2-R/AGC AGT GGA AAA GCAT GTG G; CoV-2-P/FAM-CAT AGA CAA CAG GTG CGC TC-MGBEQ [15]. Real-time RT-PCR was conducted in duplicate using the CFX96 Touch Deep Well Real-Time PCR System (Applied Biosystems). Thermal cycling conditions were 50 °C for 30 min (reverse transcription phase); 95 °C for 10 min (inactivation of the RT phase); 95 °C for 15 s; and 60 °C for 45 s (45 amplification cycles). 

For RT-PCR, we used the nuclease-free water supplied by the kit as a negative control and an aliquot of the previously extracted FCoV as a positive control.

Cycle threshold (Ct) cutoffs for RT-PCR were used as indicators of the SARS-CoV-2 RNA copy number in the samples, with lower Ct cutoffs corresponding to higher viral copy numbers. A Ct value < 40 was interpreted as positive for SARS-CoV-2 RNA.

### 2.4. Statistical Analyses

We carried out statistical analyses using Fisher’s exact test to compare the different percentages of positivity among swabs with respect to the type of surface analyzed, the category of surface analyzed, and the COVID-19 risk level where each supermarket was located (color coded) to detect any statistically significant differences. 

Multivariate analysis using the Poisson Regression Model was done to compare results of number of PCR cycles about SARS-CoV-2 surface contamination with respect to the type of surface analyzed, the COVID-19 risk level where each supermarket was located (color coded), and overall data on COVID-19 cases found 14 days before and 14 days after the sampling date in the province of analysis. The choice of the 14 days before and after was identified in such a way as to consider all the possible cases of infection present in the examination area and in the sampling period on the basis of the maximum incubation period of the infection. The data of COVID-19 cases were collected, as well as time of day that the sample was collected [16]. 

Descriptive parameters were transformed into numbers (ordinal encoding technique), assigning consecutive numbers to each parameter in alphabetical order (e.g., Parameter A = 1, Parameter B = 2, and so on) [17]. To standardize the different units of measurement for the six independent parameters, the data were normalized using the following formula [18]: x normalized = (x − xminimal)/(xmaximum − xminimal); a Poisson regression model was used. All the above parameters were considered in the preliminary model. The possible final model included only those parameters with a *p*-value < 0.05. To quantify the possible effects of the above parameters on the PCR trend, we calculated the effect (eβ − 1), which corresponds to the relative risk. 

In both types of analysis, a statistically significant result was considered with *p*-values < 0.05. We used R version 3.6.3 in the statistical analysis (The R Project for Statistical Computing, Vienna, Austria).

## 3. Results

A total of 300 surface swabs were tested; of these, 13 (4.3%) swabs were positive for SARS-CoV-2 RNA. The shopping trolley handle and scales were the most frequently contaminated among all surface categories (5.0%, respectively), followed by refrigeration system handles (3.8%), and keyboards (3.8%). Among sampling points, ice cream freezer handles and mixed-product freezer handles were the most frequently positive for SARS-CoV-2 RNA (14.3%, respectively), followed by shopping trolley handles (10%) and self-service produce scales (5.9%) (Table 1). 

The comparison of positive RT-PCR test results between shopping trolley handles and push trolley handles showed a significant difference (10.0% vs. 0.0% *p* = 0.03). The difference in proportion of positives between mixed-product freezer handles (14.3%) and push trolley handles (0%) was also significant (Fisher’s exact test, *p* = 0.034) (Table 2). Multivariate analysis using Poisson’s regression did not lead to statistically significant results with the preliminary model. In the 13 positive samples, we found an average Ct of 37.51, with a median Ct of 37.30 (range 36.88–38.63).

According to COVID-19 risk level, 8/180 (4.4%) samples were positive from supermarkets located in areas at the orange level and 5/120 (4.2%) samples from those located in red-level zones. No significant difference was observed between swab positivity among supermarkets in areas at different risk levels (*p* = 1.00).

## 4. Discussion

It is scientifically proven that surfaces contaminated with viruses and bacteria can act as a medium for the transfer of microorganisms to the hands, and subsequently, to the gastrointestinal or respiratory tract [19]. With respect to SARS-CoV-2, it has been widely demonstrated that infection with this virus mainly occurs via the respiratory tract [2,3]. However, contact of mucous membranes with a contaminated surface and/or with droplets can also lead to infection with SARS-CoV-2 [12]. There are many opportunities for environmental contamination (air and surfaces) with SARS-CoV-2 in hospital settings where COVID-19 patients may be present [4,7,8]. On the contrary, evidence regarding environmental spread of SARS-CoV-2 in the community is lacking. Pitol et al. [20], evaluated that the risks of community transmission of SARS-CoV-2 through surfaces are low and that the effectiveness of surface disinfection is highly dependent on the infection prevalence and the frequency of contacts. Although it is estimated that the transmission of SARS-CoV-2 via fomites is low, it is possible and can favor the onset of new cases during outbreaks.

In our study, we examined different surfaces in supermarkets during periods of limited mobility owing to COVID-19 restrictions. We found the presence of SARS-CoV-2 RNA contamination in 4.3% of the swab samples analyzed.

These results are consistent with other papers in which less than 10% of the swabs were contaminated by SARS-CoV-2 [10]. No significant difference between supermarkets in areas at the two highest (red and orange) risk levels were found. A potential hypothesis to explain this result can be due to the lack of period between the contamination of SARS-CoV-2 on surfaces and the real onset of infection or disease.

It is important to emphasize that entering supermarkets at these risk levels involved the obligatory use of masks and disinfection/sanitization practices. However, we found that freezer handles and trolley handles were frequently positive in testing for SARS-CoV-2 RNA contamination.

The highest contamination of these surfaces is probably related to the occurrence of use. In fact, they were frequently touched by customers and were all non-porous surfaces with the same ability to retain contamination of SARS-CoV-2. Regarding the shopping trolleys, we observed a significant contamination of the trolley handles. This result can be correlated with the availability of dispensers for hand sanitation. In our study, in all supermarkets, the hand hygiene dispenser was positioned near the push trolley deposit at the entrance to the supermarket, and the other shopping trolleys were in areas diametrically opposite to the disinfectant dispenser. We can assume that although it was strongly recommended to sanitize hands, not all customers disinfected their hands at the entrance, but rather they immediately went to take the shopping trolleys. Therefore, customers using the push shopping trolleys were more likely to disinfect their hands before using the shopping trolleys because the dispenser was near. These data lead us to underline the importance of more careful sanitization on these surfaces during the cleaning and disinfection procedures. This is essential hand hygiene, so it is necessary to increase the number of sanitation systems in community environments, in particular in proximity to surfaces that are the most frequently touched.

Scientific evidence has shown that the presence of SARS-CoV-2 RNA on a surface does not mean that the surface itself is infectious. We found Ct values between 36.88 and 38.63 in our positive samples, i.e., tending toward high values; however, some studies have shown that high values are indicative of partial RNA degradation or low viral load [21,22]. Consequently, we can hypothesize that, despite compliance with precautionary measures, SARS-CoV-2 RNA can circulate in enclosed environments frequented by healthy, perhaps asymptomatic, individuals. These observations do not mean that the surfaces contaminated by SARS-CoV-2 RNA represent a high danger of transmission of the virus, in fact the results of our study cannot irrefutably support these theories but can only show the presence of SARS-CoV-2 genetic material, not how much this can be correlated to the danger of infection.

The virus can survive on surfaces made of various materials and its degree of infectivity can vary from hours to days, depending on the environmental conditions; environments with high temperatures and high humidity can inactivate the virus more quickly [23,24]. SARS-CoV-2 has been shown to survive for up to 4 h on copper surfaces, for up to 24 h on cardboard, and for up to 2–3 days on plastic and stainless steel [11,25]. In China, COVID-19 clusters have been repeatedly linked to imported frozen raw foods. SARS-CoV-2 RNA has been detected on food packaging materials (frozen shrimp packaging), food storage environments (shipping containers), and even some foods (frozen chicken wings) [3]. In this study, we did not analyze the type of material at each sampling point. We were more interested in verifying whether this virus could be found in environments that are frequented by the public during periods of restricted mobility in the COVID-19 pandemic. In terms of both significant and non-significant data, our findings suggest interesting conclusions. We further confirmed that surfaces of public environments can be contaminated by SARS-CoV-2 RNA and we hypothesize that they could be a potential virus transmission vehicle. It is notable that more frequently positive surfaces were those hypothesized to be most often touched by hands. Thus, we highlight the need to pay particular attention to hand and environmental hygiene.

Detection of SARS-CoV-2 on surfaces believed to be touched frequently and by many people in a community indicates that increased sanitization and hand hygiene in public spaces, such as grocery stores, may be useful in decreasing SARS-CoV-2 transmission via fomites.

During periods with high circulation of SARS-CoV-2 RNA, it is necessary to intensify cleaning management programs, such as by installing disinfectant solution dispensers near high-contact surfaces used by the public [26]. Furthermore, to continue drawing attention to the need for adopting adequate preventive procedures, it is necessary to increase the number of graphic signs in public places, as well as repeated broadcast messages [27].

With a view to the protection and safety of employees, adoption of a specific protocol to avoid the spread of SARS-CoV-2 RNA is needed in stores frequented by large numbers of people each day [26]. This is in addition to recent recommendations of the Italian Institute of Health [28].

This study has some limitations. First, we did not isolate the virus from samples to assess viability; we also did not collect air samples or test supermarket customers. Furthermore, more in-depth multivariate analysis of factors with non-significant results is warranted, as well as including other parameters (e.g., size of POS devices, frequency of POS use, cases occurring specifically in the municipalities where sampled supermarkets are located). In light of the emergence of new viral variants, this is our future aim, using more standardized environmental detection techniques. 

Moreover, the introduction of quantitative methodologies to search for SARS-CoV-2 on swab samples is another possible development of our research. This methodology would allow us to apply a QMRA model [10] to estimate infection risk of the disease on every surface. 

## 5. Conclusions

Our study shows that surfaces in non-hospital environments can be contaminated by SARS-CoV-2 RNA. Contamination in public spaces can occur and warrants further investigation to evaluate viability and risk, including collection of behavioral data to understand what surfaces are touched the most often, how often people hand sanitize, and how often they touch their face. Considering that surfaces such as trolleys with handles have been found to be more often contaminated, perhaps it would be appropriate to pay more attention to these surfaces during the sanitization procedures. Certainly, it remains essential to underline the importance of prevention measures such as ventilation of environments, careful hand sanitation, and correct usage of masks in public locations in the community, such as supermarkets, and commensurate with the number of visitors at such locations.

## Figures and Tables

**Table 1:** Analyzed samples, distributed according to category and surface type. In bold we indicate the total positive samples for category.

Category (*n*)	Surface (*n*)	Positive Samples *n* (%)
**Shopping trolley handles (120)**		**6 (5.0)**
	Trolley handles (60)	6 (10.0)
	Push trolley handles (60)	0
**Scales (20)**		**1 (5.0)**
	Self-service produce scales (17)	1 (5.9)
	Self-service bread scales (3)	0
**Refrigeration system handles (80)**		**3 (3.8)**
	Refrigerator handles (23)	0
	Freezer handles (57)	3 (5.3)
**Keyboards (80)**		**3 (3.8)**
	Cash register keyboard used by cashiers (38)	2 (5.3)
	POS keyboard used by customers (42)	1 (2.4)
**Total (300)**		**13 (4.3)**

**Table 2:** Summary of statistically significant results of Fisher’s exact test.

Comparison (*n*° of Samples)	Proportion of Positive to SARS-CoV-2	Fisher’s Exact Test Results
shopping trolley handles (60) *vs*. Push trolley handles (60)	11.1% *vs*. 0%	*p* = 0.03
mixed-product freezer handles (14) *vs*. push trolley handles (60)	14.3% *vs*. 0%	*p* = 0.034

## Data Availability

Not applicable.
